# *In situ* Biological Dose Mapping Estimates the Radiation Burden Delivered to ‘Spared’ Tissue between Synchrotron X-Ray Microbeam Radiotherapy Tracks

**DOI:** 10.1371/journal.pone.0029853

**Published:** 2012-01-06

**Authors:** Kai Rothkamm, Jeffrey C. Crosbie, Frances Daley, Sarah Bourne, Paul R. Barber, Borivoj Vojnovic, Leonie Cann, Peter A. W. Rogers

**Affiliations:** 1 Health Protection Agency Centre for Radiation, Chemical and Environmental Hazards, Chilton, Oxon, United Kingdom; 2 Gray Institute for Radiation Oncology and Biology, University of Oxford, Oxford, United Kingdom; 3 The University of Melbourne Department of Obstetrics and Gynaecology, The Royal Women's Hospital, Parkville, Victoria, Australia; 4 William Buckland Radiotherapy Centre, Alfred Hospital, Melbourne, Victoria, Australia; 5 Victorian Cancer Biobank, Southern Health, Monash Medical Centre, Clayton, Victoria, Australia; Mizoram University, India

## Abstract

Microbeam radiation therapy (MRT) using high doses of synchrotron X-rays can destroy tumours in animal models whilst causing little damage to normal tissues. Determining the spatial distribution of radiation doses delivered during MRT at a microscopic scale is a major challenge. Film and semiconductor dosimetry as well as Monte Carlo methods struggle to provide accurate estimates of dose profiles and peak-to-valley dose ratios at the position of the targeted and traversed tissues whose biological responses determine treatment outcome. The purpose of this study was to utilise γ-H2AX immunostaining as a biodosimetric tool that enables *in situ* biological dose mapping within an irradiated tissue to provide direct biological evidence for the scale of the radiation burden to ‘spared’ tissue regions between MRT tracks. Γ-H2AX analysis allowed microbeams to be traced and DNA damage foci to be quantified in valleys between beams following MRT treatment of fibroblast cultures and murine skin where foci yields per unit dose were approximately five-fold lower than in fibroblast cultures. Foci levels in cells located in valleys were compared with calibration curves using known broadbeam synchrotron X-ray doses to generate spatial dose profiles and calculate peak-to-valley dose ratios of 30–40 for cell cultures and approximately 60 for murine skin, consistent with the range obtained with conventional dosimetry methods. This biological dose mapping approach could find several applications both in optimising MRT or other radiotherapeutic treatments and in estimating localised doses following accidental radiation exposure using skin punch biopsies.

## Introduction

Microbeam radiotherapy (MRT) uses an array of microplanar radiation beams, produced by collimating synchrotron X-rays, to irradiate the biological target with beams of typically dozens of micrometres width which are separated by a few hundred micrometres (reviewed in [1], [2]). Rather than fractionating the total treatment dose in time to allow normal tissues to recover, as in conventional radiotherapy, MRT utilises spatial fractionation to spare normal tissues which are surprisingly resistant to single acute doses of hundreds of gray (e.g. [3], [4]). Conversely, tumours, despite being only partially exposed to a high radiation dose, appear to be sensitive to MRT treatment in both unidirectional and interlaced regimes, and can be controlled under conditions where normal tissues are less damaged than with conventional broad beam radiotherapy (e.g. [5], [6]). Possible explanations for these intriguing characteristics include differential microvascular repair in normal tissues vs. tumours [7]–[9] and the bystander effect/cellular communication [6], [10]. As yet, however, no conclusive data have emerged to fully support or refute these proposed effects.

One important parameter in MRT is the peak-to-valley dose ratio (PVDR). It indicates how much radiation is given to the ‘spared’ tissue in a dose valley between two microbeams relative to the peak dose in the centre of the beam. This parameter is extremely important to help understand the clinical response to different MRT conditions and is a prerequisite for systematically optimising treatment regimens; yet its experimental measurement with radiation dosimeters or its mathematical calculation using Monte Carlo codes for radiation transport processes is very complex and associated with large uncertainties (e.g. [11]–[15]).

Immunocytochemical detection of the phosphorylation of the histone H2AX to form γ-H2AX has been used extensively to visualise and quantify ionising radiation-induced DNA double-strand breaks (reviewed in [16], [17]). Unlike any other current method of measuring DNA damage, γ-H2AX combines a number of useful features: excellent sensitivity, *in situ* detection, simple quantification, large dynamic range, single cell analysis and the possibility to combine this marker with other immunocytochemical or histological techniques. These features have led to its use as a qualitative marker for mapping radiation damage in MRT-treated cell cultures or tissues [6], [18], [19]. Here we have used γ-H2AX immunostaining not only to visualise MRT tracks but to quantify radiation-induced DNA damage between those tracks in cultured fibroblasts and murine skin sections. Foci counts in these valleys were compared with a calibration curve for γ-H2AX foci induction using known broad beam synchrotron X-rays doses to estimate doses delivered as a function of distance from the nearest MRT track and determine PVDR values *in situ*.

## Materials and Methods

### Ethics statement

All animal work was approved by the animal welfare committees of the Japanese Synchrotron Radiation Research Institute (SPring-8 approval ID 2007A1750) and Monash University (approval ID MMCA 2007/29). Animals were anaesthetized for all procedures.

### Fibroblast cultures

Human MRC-5 foetal lung fibroblasts (obtained from ATCC, #CCL-171) were cultured in EMEM with 2 mM glutamine, 1% non essential amino acids and 10% foetal bovine serum (FBS) at 37°C in a humidified incubator at 5% CO_2_. Cells were grown to confluence and irradiated in Nunc Flasks on Slides and returned to the incubator immediately after irradiation.

### Mouse preparation

Adult, female BALB/c mice were fed a standard rodent diet and given water ad libitium in the animal house of the biomedical imaging centre on the SPring-8 synchrotron campus. The animals were anaesthetized using a 1∶10 dilution of pentobarbitone (Nembutal, Sigma Aldrich, St Louis, MO, USA) at a concentration of approximately 1 ml/kg (equivalent to 50 mg/kg body weight). The hair along the animals' back was shaved using a standard electric rodent shaver. A skin flap was raised along the animals' back and pre-cut lengths of radiochromic film (Gafchromic HD-810, ISP Corp, New Jersey, USA) were placed on both sides of the skin flap to identify the entrance and exit radiation fields. The mice were secured on a perspex jig by placing them inside a 50 ml vial with a cut-away made to allow the skin flap to be raised for irradiation [6].

### Microbeam and broadbeam irradiations

All irradiations were carried out on beamline BL28B2 at the SPring-8 synchrotron, Hyogo, Japan, using a similar setup as described previously [6], [11]. A bending magnet produced polychromatic X-rays from electrons travelling at relativistic speeds around a storage ring with energies of 8 GeV and a stable beam current of approximately 100 mA. A fixed geometry, tungsten/kapton collimator [12] was used to segment the incident, broad beam into an array of quasi-parallel horizontal, planar microbeams. The thickness of the collimator in the direction of the beam was approximately 5 mm. The nominal microbeam width was 25 µm, with a center-to-center spacing of 200 µm. The vertical height of the incident, broad beam was 1 mm, producing five micro-planar beams post-collimation. The exposure time was adjusted by varying the opening time of an upstream lead shutter, which blocked the X-ray beam. For the MRT treatments, copper filters of 11.5 mm thickness were used to reduce the in-air entrance dose rate from approximately 80 Gy/s to approximately 4 Gy/s, to enable the precise delivery of low doses within the mechanical capabilities of the shutter. The mean energy of the microbeam X-rays was 162 keV. For the broad beam calibration studies, a total of 15.75 mm Cu, 7 mm Al, and 1 mm Fe were used to reduce the dose rate to about 1.5 Gy/s. The mean X-ray energy for the broadbeam studies was 175 keV.

One group of mice (n = 3) were irradiated with uni-directional MRT with the beam geometry described above, using different in-beam entrance doses (3.9, 7.8, 13.7, 19.6, 48.8, and 97.5 Gy) in six adjacent fields on the raised skin flap. A further group of mice (n = 3) were irradiated with a range of broad beam radiation entrance doses (0.15, 0.45, 1.35, 1.8, 2.7, 3.6, and 4.5 Gy). Three mice were used as sham-irradiated controls. These un-irradiated mice were prepared, anaesthetized and positioned on the treatment jig in an identical fashion to the irradiated mice. Immediately post-irradiation, a fine-gauge needle containing Evan's Blue dye (Sigma Aldrich, St Louis, MO, USA) was used to mark the corners of the irradiated fields on the mice skin using the exposed radiochromic film as a visual guide. The mice recovered from the anaesthesia and were returned to the animal facility in the biomedical imaging centre at SPring-8. Using the same beam geometry as above, fibroblast cultures in flask slides were irradiated in duplicates with uni-directional MRT and broad beam X-rays using different in-beam entrance doses (8, 10, 20, 50 and 100 Gy for MRT and 0.45, 0.9, 1.8, 2.7, 3.6 and 4.5 Gy for broad beam). Again, sham-exposed controls underwent the same treatment as exposed flask slides.

### Fibroblast fixation, immunofluorescence staining and γ-H2AX foci scoring

Following MRT treatment and repair incubation of 30 min or 2 h, fibroblast cultures in Nunc Flasks on Slides were fixed with 3.7% formaldehyde in phosphate-buffered saline (PBS) for 15 min, filled up with PBS and posted from Japan to the UK for immunostaining and analysis. Permeabilisation with Triton X-100, blocking and immunofluorescence staining with γ-H2AX antibody clone JBW301 (Upstate, Charlottesville, VA, USA) and AlexaFluor488-conjugated anti-mouse secondary antibody (Invitrogen) were performed as previously described [20]. An automated microscopy system with a 100× lens (1.3NA) and Hamamatsu ORCA-ER camera was used for acquiring z-stacks of 8 sequential focus planes in 0.5 µm steps per field of view and TRI2 software (version 2.2) was used for foci scoring in maximum intensity projection images and for spatial mapping of cells relative to MRT tracks [21].

### Tissue harvesting, processing and immunohistochemistry for γ-H2AX

Mice were culled by cervical dislocation at 30 min post-irradiation. The skin flap was excised from the mouse, flattened and fixed in formalin for at least six hours. Small sections of irradiated tissue were removed with the cut surface oriented perpendicular to the path of the microbeams. These were placed into histology cassettes and returned to Australia in phosphate-buffered saline for subsequent dehydration and embedding into paraffin. Blocks of paraffin-embedded skin were then sent to the UK for sectioning, immunohistochemical staining and analysis. Four-micrometre sections of formalin-fixed paraffin-embedded skin were deparaffinized, rehydrated and microwaved at 850 W for 4 min in 10 mM citric acid for antigen retrieval. Slides were stained in a DAKO autostainer using ChemMate EnVision Detection Kit Peroxidase/DAB, Rabbit/Mouse (K5007, DAKO UK Ltd., Ely, UK) and anti-γ-H2AX antibody at 2.5 µg/ml (clone JBW301, Upstate, Charlottesville, VA, USA). Slides were weakly counterstained with haematoxylin. γ-H2AX foci per nucleus were scored by eye using bright field microscopy at ×1000 magnification and the individual distance of each nucleus to the closest microbeam track was measured using Motic Images 2.0 software on digital images obtained with a 20× objective and Moticam 2500 camera (Motic, Wetzlar, Germany).

## Results

**Figure 1:**
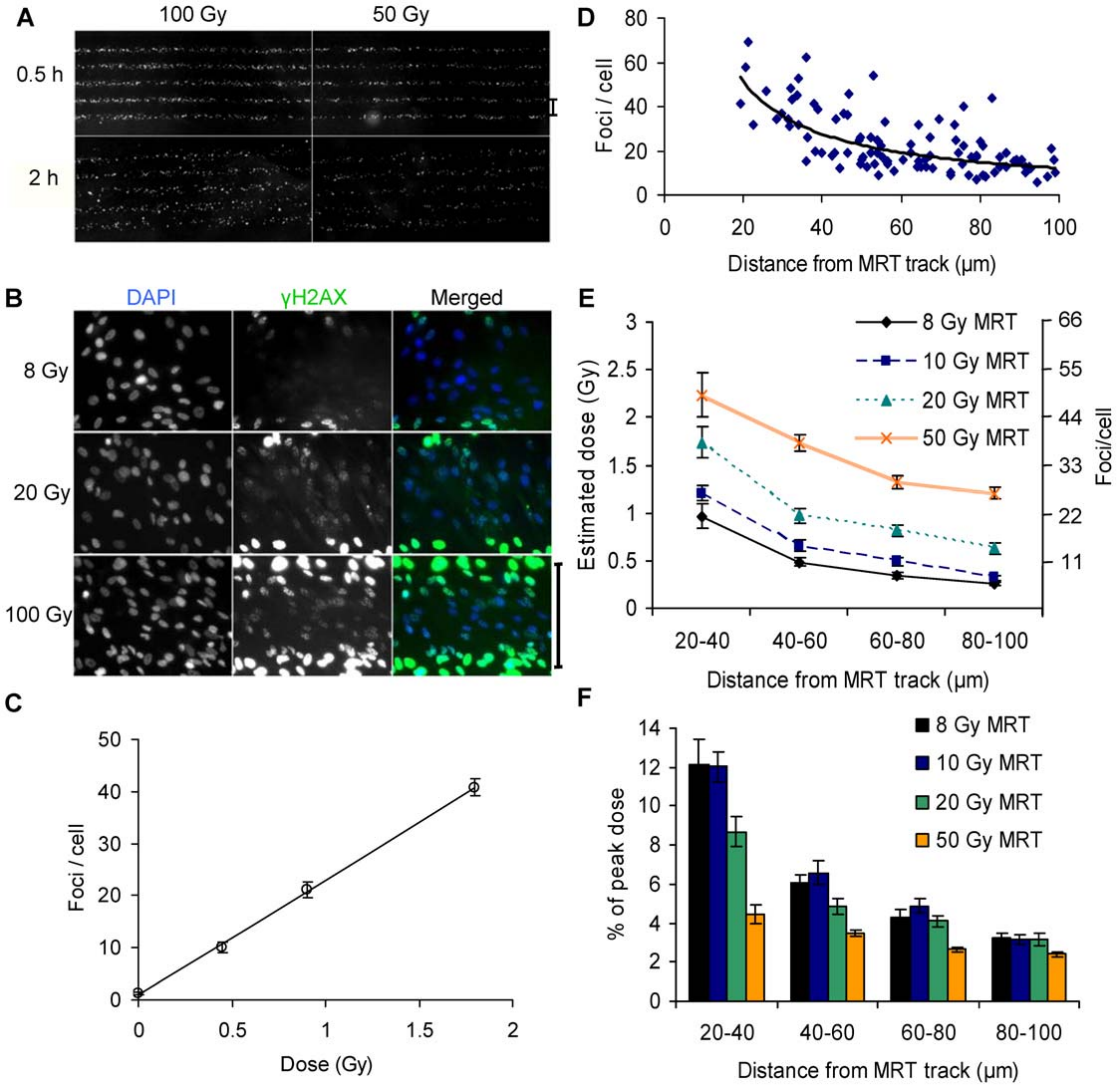
γ-H2AX immunofluorescence analysis of human fibroblast cultures following microbeam radiotherapy. (**A**) ‘Stripes’ of γ-H2AX-stained cells are visible 30 min (top) and 2 h (bottom) after treatment with peak doses of 100 (left) and 50 Gy (right). (**B**) High magnification images of cells in peak and valley regions 30 minutes after treatment with different peak doses. Scale bars in (A) and (B) are 200 µm. (**C**) Dose response curve for foci induction 30 minutes after broadbeam synchrotron X-irradiation. The line shows a linear regression fit (y = 22.1x+0.9; R^2^>0.99). (**D**) Foci levels in valley regions as a function of distance of the centre of the cell nucleus from the centre of the nearest microbeam track with a peak dose of 20 Gy. Each data point represents one cell. The curve was fitted to guide the eye. (**E**) Calculation of radiation doses delivered to valleys between microbeam peak doses of 8, 10, 20 and 50 Gy, based on the comparison of γ-H2AX foci levels in valleys with the calibration curve in panel C obtained for broadbeam-irradiated cultures. (**F**) Percentage of the peak dose as a function of distance from the centre of the nearest microbeam track. Error bars are the standard deviation from the analysis of two separately irradiated cultures. Error bars for dose estimates ignore uncertainties associated with the calibration curve.

Human MRC-5 foetal lung fibroblast cultures in slide flasks were fixed and immunostained for γ-H2AX after 0.5 and 2 hour incubations following exposure to different doses of micro-planar beams of synchrotron X-rays. ‘Stripes’ of bright γ-H2AX immunofluorescence staining were observed which mirrored the spatial distribution of the microbeams, as determined using radiochromic films on the same samples (Figure 1A). The intensity and width of the γ-H2AX stripes increased with dose. In contrast to a well defined stripe pattern seen with the 0.5 hour samples, fibroblasts incubated for 2 hours showed a less defined geometrical pattern, likely caused by increased cell movement during the longer post-irradiation incubation period. All subsequent biodosimetric analyses of fibroblast cultures were performed using the 0.5 hour samples, to minimise artefacts associated with cell migration. At higher magnification, foci were visible in cells located in the ‘valleys’ between microbeam tracks (Figure 1B). Their abundance increased with peak dose, and at the highest peak dose of 100 Gy only cells in the centre of the valleys, i.e. as far away from the tracks as possible, showed discernible foci whilst cells closer to the tracks showed almost pan-nuclear γ-H2AX staining, as did the cells inside the tracks.

In order to calculate valley doses using γ-H2AX foci as a biodosimeter, a calibration curve was established using the same irradiation conditions. To this end, fibroblast cultures in slide flasks were exposed to different doses of broadbeams of the same synchrotron X-rays that were used for microbeam irradiations. Following incubation at 37°C for 0.5 hours, samples were fixed and immunostained for γ-H2AX. Z-stacks of fluorescence images were acquired and analysed using the ‘object count’ module of the *Tri2* software [21]. Foci levels increased linearly with dose for doses up to 1.8 Gy (Figure 1C), and a linear regression fit produced a yield of 22.1±0.4 foci per cell per gray with a background level of 0.9 (R^2^ = 0.99). At higher doses, foci levels saturated, probably due to increasing signal overlap (data not shown); these samples were therefore excluded from the analysis.

The same image acquisition and analysis process was performed with MRT-treated fibroblasts. Foci levels per cell were analysed as a function of the distance of the centre of each cell nucleus from the nearest MRT track, using the spatial information provided by the *Tri2* software. Considerable cell-to-cell variation but overall declining foci levels with increasing distance were observed for all doses; see Figure 1D for an example showing data for 20 Gy MRT peak dose. To obtain average damage levels for conversion into dose, cells in valley regions between microbeam tracks were grouped into four distance categories (20–40, 40–60, 60–80 and 80–100 µm from the centre of the nearest microbeam track). Figure 1E shows a very consistent relationship between relative spatial position and foci levels for all the analysed MRT peak doses ranging from 8 Gy to 50 Gy. These foci levels could be converted to doses using the calibration curve in Figure 1C and are shown as percentage of the peak dose for each irradiation condition in Figure 1F. This indicates a dose-independent maximum peak-to-valley ratio of 30–40 for the central valley region between two microbeam planes. Foci levels at 100 Gy peak dose were too high for reliable scoring.

**Figure 2:**
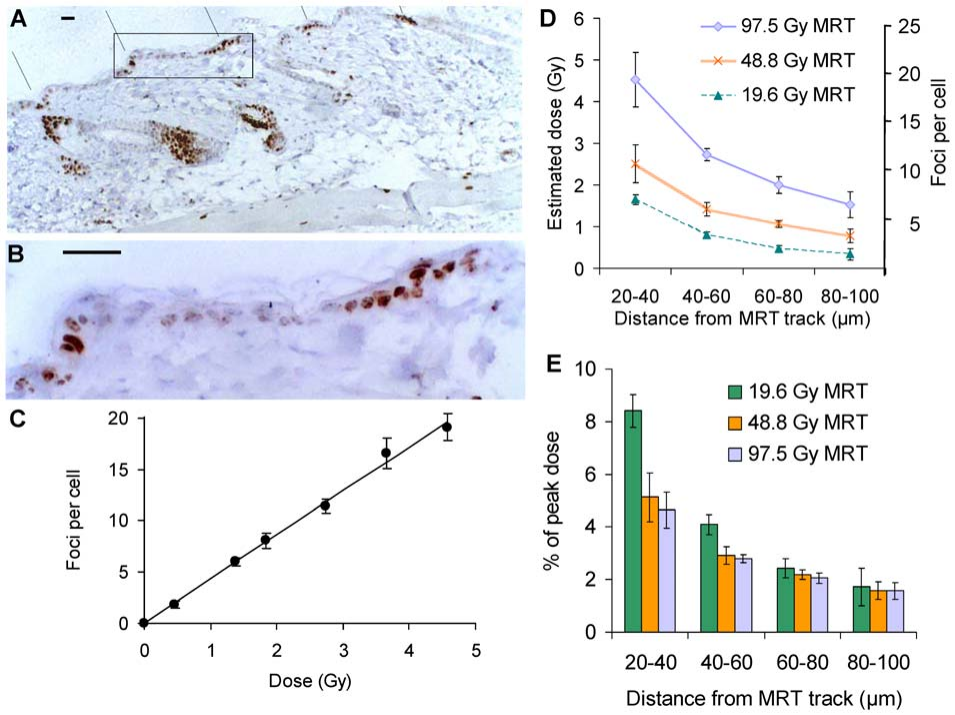
Immunohistochemical analysis of γ-H2AX in murine skin sections excised 30 min after microbeam radiotherapy. (**A**) Stripes of γ-H2AX-stained cells show the position and angle (from 11 o'clock, as indicated with the black lines) of MRT tracks with a peak dose of 97.5 Gy. (**B**) High magnification image of the region indicated with a box in (A). Scale bars in (A) and (B) are 25 µm. (**C**) Dose response curve for foci induction 30 minutes after broadbeam synchrotron X-irradiation. The line shows a linear regression fit (y = 4.3x+0.05; R^2^>0.99). (**D**) Calculation of radiation doses delivered to valleys between microbeam peak doses of 19.6, 48.8 and 97.5 Gy, based on the comparison of γ-H2AX foci levels in valleys with the calibration curve in panel C obtained for broadbeam-irradiated skin. (**E**) Percentage of the peak dose as a function of distance from the centre of the nearest microbeam track. Error bars are the standard deviation from the analysis of skin sections from three animals. Error bars for dose estimates ignore uncertainties associated with the calibration curve.

To quantify dose distributions in irradiated tissues, skin sections from mice held for 0.5 hours following exposure to different doses of microplanar beams of synchrotron X-rays were immunostained for γ-H2AX and counterstained with hematoxylin. Stripe patterns of brown γ-H2AX staining were observed (Figure 2A; incident beams from ∼11 o'clock) which did not quite match the perfectly parallel and regular patterns observed in fibroblast cultures in Figure 1. Distortion and shrinkage of skin during tissue processing and the inhomogenous distribution of cells, especially in the dermis, may have contributed to this effect. Due to these geometric distortions and the low overall cell density in skin layers below the epidermis, scoring of γ-H2AX foci as a function of distance to the closest microbeam track was only feasible for epidermal cells. As for fibroblasts, the intensity and width of the γ-H2AX stripes increased with dose (not shown).

At higher magnification, foci were visible in cells located in the valleys between microbeam tracks whereas cells close to or within the tracks showed almost pan-nuclear γ-H2AX staining (Figure 2B), as seen in Figure 1 for fibroblasts.

Again a calibration curve for foci yield versus radiation dose was established. Using the same irradiation conditions as for MRT treatments, skin flaps were exposed to different doses of broadbeam synchrotron X-rays, excised 0.5 hours later and processed using the same protocol as for the microbeam-treated mice. As automated scoring of solid-stained γ-H2AX foci in skin sections using image analysis software was not available, foci were scored manually at 1000× magnification. Whilst there was again a linear induction of γ-H2AX foci with dose, their yield per unit dose was significantly lower (Figure 2C). A linear regression fit produced a yield of 4.29±0.13 foci per cell per gray with a background level of 0.05 (R^2^ = 0.99).

For MRT-treated skin, γ-H2AX foci were scored in the same way by eye and the distance of each cell nucleus to the nearest MRT track was measured using the Motic Images 2.0 software. Average foci levels and corresponding dose estimates for cells grouped into distance categories relative to the nearest MRT track are shown in Figure 2D for three different MRT peak doses. The foci yields and dose estimates as a function of the distance from the nearest microbeam have a similar profile to the data obtained for fibroblasts (Figure 1E), and absolute dose estimates are very similar, despite the considerable difference in absolute foci yields per unit dose determined for the two different sample types and methodological approaches used. Again the percentage of the peak dose delivered to valley regions between tracks had a similar minimum level furthest away from the MRT tracks (Figure 2E), resulting in a maximum peak-to-valley dose ratio of approximately 60, somewhat higher than what had been observed for cell cultures on slides (Figure 1F).

## Discussion

The γ-H2AX foci yield of 22.1 per cell per gray at 0.5 hours after irradiation observed in fibroblasts in this study is similar to yields of 20–25 per cell per gray at 0.25 hours after irradiation reported in previous studies where fibroblasts were X-irradiated on slides [20], [22]. In contrast, the foci yield of only 4.3 per cell per gray measured in skin sections is considerably lower. A number of factors have probably contributed to this effect. Antigen masking due to extended incubation in formalin was addressed by boiling skin sections in citric acid but may still have resulted in reduced antibody binding. Sectioning at 4 µm thickness conceivably cut off parts of nuclei which are normally bigger in diameter, thus reducing the average amount of DNA available for γ-H2AX staining. Solid-stained γ-H2AX foci are not as crisp and easy to score as immunofluorescence-stained foci, partly because of masking effects of the hematoxylin counterstain which was nonetheless necessary – but used at a low staining intensity – to enable reliable detection of cell nuclei. Together, these factors may explain the five-fold discrepancy between foci yields in fibroblast cultures and skin sections obtained in this study. Nonetheless, this study confirms previous work indicating that chromogenic immunohistochemical staining can be used to quantify ionising radiation-induced foci in tissue sections [23], [24].

The extensive movement of fibroblasts in culture observed as early as 2 hours after MRT treatment is consistent with the recently reported considerable tumour – but not skin – cell movement *in vivo* [6]. To minimise distortion of the original radiation geometry and associated flattening of the PVDR by cell movement, cell cultures were incubated for only 30 min post-irradiation, to allow γ-H2AX foci to form, yet minimise cell movement. However, even in these samples it is possible that PVDRs have decreased slightly as a consequence of limited cell movement. This may in fact be one reason for the slightly lower PVDR values obtained for fibroblast cultures (30–40) compared to skin sections (approximately 60) for the same MRT beam geometry.

Nonetheless, it is reassuring that spatial distributions of foci measured across valleys between microplanar beams were very consistent and that PVDRs were still fairly compatible for these two entirely distinct experimental setups, despite vastly different absolute foci yields obtained by automated scoring in immunofluorescence-stained fibroblast cultures and by manual scoring in solid-stained murine skin sections (compare Figures 1C and 2C). These PVDRs are similar to ratios measured for the same collimator with radiochromic film [11] but considerably lower than measurements using phosphor coupled to a charge-coupled device camera and Monte Carlo calculations for radiation transport [12]. It is important to note that PVDR values can differ dramatically for different collimator geometries and materials and also depend on the characteristics of the incident X-ray beam, thickness of the tissue layers traversed by the radiation, and depth of the targeted tissue layer. For the murine skin experiments the thickness of the films and the skin have to be considered, whereas for the fibroblast cultures the thickness of film and slide flask material are important This makes it difficult to compare different experimental setups, introduces considerable complexity and uncertainty about the ‘true’ value and underlines the importance of measuring dose profiles where they matter, i.e. in the targeted and surrounding tissues whose biological responses to MRT determine treatment outcome. This is exactly where γ-H2AX-based biodosimetry has its unique advantage over other dosimetric methods, because it provides direct biological evidence for the scale of the radiation burden to ‘spared’ tissue regions located between planar microbeams.

Assuming that cell movement is independent of dose, it alone cannot explain the relatively high foci levels observed for fibroblasts located close to the track at low doses. The finding of a similar effect at low doses in mouse skin where, in contrast to implanted tumours and to the fibroblast cultures studied here, no significant cell movement was observed for days following microbeam irradiation [6], also suggests that cell movement alone cannot explain this effect. Inter-cellular signalling between neighbouring cells following irradiation, causing excess chromosomal aberrations and DNA damage foci in bystander cells, could potentially explain this phenomenon and has indeed been observed in cell cultures [25]–[27], three-dimensional tissue models [28], [29] and *in vivo* (reviewed in [30]). However, methodological limitations could also have contributed. For example, human scorers as well as image analysis software-based scoring procedures tend to under-score in cells with high foci levels such as those very close to a high dose microbeam. This systematic bias would have exactly the effect observed here, although the absence of such a bias in the broad beam calibration curve, i.e. no saturation of foci yields towards high doses, argues against this notion. Additional effort will be required to identify the mechanism underlying the observed ‘anomaly’.

The relatively consistent dose estimates obtained for MRT-treated cell cultures and skin suggest that γ-H2AX foci analysis represents a fairly robust and reproducible methodology for spatially resolved *in situ* biodosimetry in biological tissues exposed to complex radiation geometries. Such biological dose maps may prove useful not only in further characterising clinically relevant parameters in microbeam radiotherapy, where e.g. decreasing PVDRs with penetration depth into the tissue could be experimentally tested, but this technique, applied to a skin punch biopsy, could potentially also be used in situations of accidental radiation exposures with highly non-uniform dose distributions where localised dose estimates could help inform clinical management of the patient.
